# Identifying attachment ruptures underlying severe music performance anxiety in a professional musician undertaking an assessment and trial therapy of Intensive Short-Term Dynamic Psychotherapy (ISTDP)

**DOI:** 10.1186/s40064-016-3268-0

**Published:** 2016-09-17

**Authors:** Dianna T. Kenny, Stephen Arthey, Allan Abbass

**Affiliations:** 1 Faculty of Arts and Social Sciences, The University of Sydney, Room 468, Building H04, Sydney, 2006 Australia; 2 Melbourne Centre for ISTDP, Glen Iris, Australia; 3 Centre for Emotions and Health, Dalhousie University, Halifax, NS Canada

**Keywords:** Attachment, Music performance anxiety, Intensive Short Term Dynamic Psychotherapy, Professional musicians

## Abstract

**Introduction:**

Kenny has proposed that severe music performance anxiety that is unresponsive to usual treatments such as cognitive-behaviour therapy may be one manifestation of unresolved attachment ruptures in early life. Intensive Short-Term Dynamic Psychotherapy specifically targets early relationship trauma. Accordingly, a trial of Intensive Short-Term Dynamic Psychotherapy with severely anxious musicians was implemented to assess whether resolution of attachment ruptures resulted in clinically significant relief from music performance anxiety.

**Methods:**

Volunteer musicians participating in a nationally funded study were screened for MPA severity. Those meeting the critical cut-off score on the *Kenny Music Performance Anxiety Inventory* were offered a trial of Intensive Short-Term Dynamic Psychotherapy. In this paper, we present the theoretical foundations and rationale for the treatment approach, followed by sections of a verbatim transcript and process analysis of the assessment phase of treatment that comprised a 3-h trial therapy session.

**Case description:**

The ‘case’ was a professional orchestral musician (male, aged 55) who had suffered severe music performance anxiety over the course of his entire career, which spanned more than 30 years at the time he presented for treatment following his failure to secure a position at audition.

**Discussion and evaluation:**

The participant was able to access the pain, rage and grief associated with unresolved attachment ruptures with both parents that demonstrated the likely nexus between early attachment trauma and severe music performance anxiety.

**Conclusion:**

Intensive Short-Term Dynamic Psychotherapy is a potentially cost-effective treatment for severe music performance anxiety. Further research using designs with higher levels of evidence are required before clinical recommendations can be made for the use of this therapy with this population.

## Background

Kenny (2009, 2011) has identified three possible subtypes of music performance anxiety—(1) focal anxiety associated with realistically highly anxiety-provoking situations such as auditions and solo performances with little generalized anxiety to other situations; (2) performance anxiety associated with a comorbid diagnosis of social anxiety (social anxiety disorder); and (3) performance anxiety associated with severe, performance-impairing anxiety, co-occurring with panic and either pervasive dysthymia, dysphoria or depression. Kenny hypothesized that an unresolved attachment disorder was implied in the majority of subtype 3 and may be implicated in subtype 2 to a lesser extent, with attachment ruptures occurring later in childhood than in subtype 3. Subtype 3 has been referred to as a disorder of the self (Kohut 1971, 1977, 1984; Kohut and Wolf 1978), pre-verbal trauma (Winnicott 1945, 1965, 1974); (reactive) attachment disorder (Fonagy and Target 1997; Halpern 2004; Janus 2006; Mills 2005; Wallin 2007) and fragile character structure (Davanloo 1990, 2005).

Intensive Short-Term Dynamic Psychotherapy (ISTDP) is a short term psychotherapy that shares with other short term psychotherapies a number of common features, which include time-limited contracts, maintaining a therapeutic focus (as opposed to the free association of psychoanalysis), active therapist involvement (as opposed to the non-intrusiveness of analysts), and the use of the transference relationship involving the Triangle of Conflict (feelings/impulse, anxiety and defence) (Ezriel 1952) and the Triangle of Person/Time (past relationships, usually parents, therapist and current relationships) (Menninger and Holzman 1973) to maintain the therapeutic focus (Davanloo 1990, 2005). For a detailed explanation, see Kenny (2011).

The theoretical structure of ISTDP draws on Freudian psychoanalysis (Freud 1933), attachment theory (Bowlby 1988; Schore 2003), and the short-term psychotherapies (Malan 1979). The core therapeutic action in ISTDP is the “patient’s actual experience of his true feelings about the present and the past” (Davanloo 1990, p. 2). Davanloo (1990, 2005) developed a technique to rapidly mobilize the unconscious therapeutic alliance (Davanloo 1987) in order to remove the major resistances to change, which were not effectively removed through interpretation alone.

Internal emotional conflicts are created through ruptures in attachment relationships in the first 8 years of life (Bond 2010; Muller 2009; Pauli-Pott and Mertesacker 2009). Many are due to chronic parental misattunement to, or lack of empathy with children’s emotional signals. The age of the child at the time the rupture first occurs, and the frequency and duration of these experiences of rupture are indicators of the severity of the attachment rupture (Bond 2010). The younger the child, the more frequently the events occur and the longer the overall duration of the events, or the more persistent and unrelieved the parental misattunement, the more severe is the attachment rupture (Beebe et al. 2010; Bowlby 1960, 1973).

The rupture in the attachment relationship causes emotional pain in the child and a retaliatory rage towards the parent(s) for causing the pain. However, because the child also loves his parent(s), he feels guilt about experiencing rage towards someone he loves. The rage, guilt, grief and love are all dissociated into symptoms and are submerged under behaviours that enable the child to continue a relationship with the parent(s).This process eventually becomes a characteristic defensive system (Winnicott 1965). Whenever the child is in a situation that has the potential for a rupture of attachment, the rage, guilt, love and pain from the initial attachment rupture is re-activated. Anxiety is experienced to block the feelings from entering conscious awareness and the defensive system is automatically triggered to keep the feelings dissociated and to avoid or alter the emotionally triggering situation (Glowinski 2011). Over time, this pattern is automatically activated in any situation that has the potential to trigger the dissociated feelings about the initial attachment rupture (Amos et al. 2011), such as an evaluative musical performance.

The anxiety over the internal emotional conflict and the defensive pattern become the psychological problems in the person’s life. Anxiety can manifest in four ways:

1. Tension in the striated muscles of the body, which is associated with a number of physical problems including fibromyalgia, pain, spasm, hyperventilation and panic (Abbass et al. 2006). In a therapeutic context, striated muscle anxiety is an indication that the person has the capacity to consciously experience the dissociated feelings related to the attachment rupture(s).
2. Smooth muscle anxiety, in which anxiety is somatised into the gut, leading to gastrointestinal symptoms including nausea, reflux, cramping and the urge to urinate and/or defecate. The striated muscles remain relaxed. Chronic smooth muscle anxiety is associated with hypertension, irritable bowel syndrome and migraine (Abbass 2005; Abbass et al. 2008).
3. Cognitive perceptual disruption (CPD). A person experiencing CPD will become confused or blank in their thoughts and/or will have disturbances in one or more of their senses when experiencing anxiety (e.g., tunnel vision, blurred vision, ringing or buzzing in the ears). Visual disturbances are most common (Davanloo 1995b). Physically, the person will appear relaxed as anxiety is not being expressed in the striated muscles, but will manifest confused thinking and not be “present” in the room. Chronic cognitive perceptual disruption is associated with neurological complaints (for which no medical cause can be found) including dizziness and fainting.
4. Conversion (Axelman 2012). Instead of becoming tense, the person will become weak in one or more limbs, experience pain in one or more areas of the body, or lose the function of one or more senses (e.g., vision). Potential medical causes must always be ruled out before concluding that the symptom is an indication of conversion.

In a therapeutic context, the experience of smooth muscle anxiety, cognitive perceptual disruption or conversion indicates that a psychological restructuring process is required before the person is capable of consciously experiencing the dissociated feelings from their attachment rupture(s). In restructuring, the person is gradually exposed to increasing levels of anxiety via graded exposure to their dissociated feelings and helped to develop and maintain a striated muscle anxiety response (Davanloo 1995a). Eventually, the patient is able to consciously experience the previously dissociated feelings without undue anxiety.

In response to anxiety, defences are automatically activated. There are three main groups of defences (Davanloo 1996); (1) *Isolation of affect* is the most adaptive defensive system. Patients are aware that they are experiencing a particular emotion, but they do not know how they are physically experiencing it. Instead of the physical experience of the emotion, patients with isolation of affect experience striated muscle anxiety; (2) *repressive defences* (Davanloo 1996). Patients with repressive defences do not recognize that they are experiencing emotions. Instead feelings are dissociated into the body. Repressive defences are linked to smooth muscle anxiety where feelings are internalized/somatised into, for example, nausea, irritable bowel syndrome, depression, headache, or conversion; (3) *projective/regressive defensive system*. Patients using this cluster of defences do not perceive that they are experiencing emotions, but rather perceive that another person is experiencing the feelings that the patient would be expected to feel. Typically, these patients manifest weepiness (tears without feelings of grief), temper tantrums, explosive discharges of affect, and confusion. This defensive system is associated with cognitive perceptual disruption (Davanloo 1995b).

The combination of anxiety type and system of defence enables each patient to be located on either the Spectrum of Neurotic Character Structure (Davanloo 1999a) comprising low, moderate, and high resistance or the Spectrum of Fragile Character Structure (Davanloo 1995b).

- *Low resistance* These patients have had secure attachment relationships for at least the first 7 years of life. Their problems are of recent onset or are mild neurotic disorders. They have no rage in their unconscious. These patients are very responsive to psychotherapy.
- *Moderate resistance* These patients have had attachment ruptures at between 5 and 7 years of age. They have character disorders and diffuse psychological symptoms, experience violent to murderous rage, guilt, and grief in their unconscious from the early attachment ruptures involving one or more figures from their early life.
- *High resistance* These patients experienced attachment ruptures in the first 2–5 years of life. They have complex character pathology and highly syntonic character resistance, with a masochistic, self-sabotaging component. These patients have intense murderous rage, guilt and grief in relation to all of their early attachment figures.
- *Spectrum of Fragile Character Structure* These patients may never have experienced an attachment bond or had their attachment bonds rupture within the first 2 years of life. They cannot withstand the impact of their unconscious feelings in the first interview and require a restructuring process where they are exposed to increasing intensities of their unconscious feelings (Davanloo 1995c). They habitually use regressive and projective defences (e.g., temper tantrums, explosive discharges of affect, self-harm, drug and alcohol misuse, dissociation, and projection).

ISTDP assists the patient to fully experience their dissociated feelings and fantasies and memories that have been dissociated with these feelings. The major interventions are applied through an over-arching framework, the central dynamic sequence (CDS) (Davanloo 1999a) that guides the therapist towards the dissociated feelings and memories. The CDS can be divided into eight overlapping stages. Each stage has definable goals that need to be achieved before progressing to the next stage. As the goals of each stage are achieved, they add to and build a complex intra-psychic and interpersonal experience during which the defences are overcome and the previously dissociated feelings enter conscious awareness. The conscious experience of these dissociated feelings triggers memories associated with early attachment ruptures, enabling these previously dissociated memories and feelings to be resolved.

### Aims

The first aim of this paper was to report on the trial application of the eight stages of the central dynamic sequence of ISTDP in the first assessment session and to evaluate the degree to which early attachment trauma was present and acknowledged. The second aim was to assess the nature of the possible relationship between the attachment trauma and this musician’s MPA.

## Methods

The study received ethical approval from The University of Sydney Human Research Ethics Committee. The participant signed an Informed Consent allowing the researchers to videorecord and transcribe the trial therapy session and subsequent therapy (reported elsewhere: see Kenny et al. 2014a) and to write up the contents for scientific purposes.

### The therapeutic intervention

There are eight key groups of interventions in ISTDP that enable the therapist and patient to achieve the goals outlined above:

- **Stage 1**: *Inquiry* Many patients are unable to give an accurate account of their problems. Inquiry begins to stir the unconscious and hence the activation of the defences.
- **Stage 2**: *Pressure* (Davanloo 1999c). Once the patient begins to resist the therapist’s attempts to understand, the therapist applies pressure to the patient’s feelings or defences to overcome defences sufficiently to allow the therapist and patient to understand the problem (Abbass and Town 2013).
- *Clarification* As the therapist applies pressure, the patient’s defences increase. The therapist points out and clarifies the patient’s defences and examines the costs of these defences. As awareness rises, the patient recognizes the costs of his defences, and begins to turn against them. This process is repeated for each observed defence.
- **Stage 3**: *Challenge* (Davanloo 1999b). Once the patient has turned against his defences, the therapist challenges the patient not to use his defences, but instead to experience the feelings that have been dissociated under resistance.
- *Head on Collision* (Davanloo 1999a) combines clarification of what is happening in the moment within the patient in relation to the therapist with challenge not to engage in defences, pointing out the cost to the patient in continuing the defensive pattern and placing the responsibility for change with the patient.
- The therapist’s structured focus on the patient’s feelings and defences creates complex feelings within the patient towards the therapist, called Complex Transference Feelings (CTF). The patient feels grateful to the therapist for his relentless efforts to free him from the suffering incurred through anxiety and defences while simultaneously feeling angry with the therapist for the relentless pressure and challenge to their long-held defensive system (Davanloo 1990, 2005).
- **Stage 4:***Transference resistance* occurs when the patient resists the therapist’s efforts to reach his feelings. Pressure, Challenge, and Head on Collision are repeatedly used to drive up CTF and to overcome defences as they are mobilized to the frontline of the patient’s resistance. Eventually, the defences are exhausted, and the CTF are consciously experienced.
- **Stage 5**: *Direct access to the unconscious*. Conscious experience of previously dissociated feelings (rage, with guilt, grief, love and pain) being mobilised brings a desire and then a fantasy of attacking the therapist. This violent-to-murderous fantasy is the actual fantasy the patient had towards his attachment figure(s) at the time of the ruptures. After the rage, guilt is experienced about the violence/murder and the body of the therapist transfers to the injured/murdered body of the attachment figure(s). Guilt and loving feelings relating to the original attachment are felt followed by grief and pain about the loss of the attachment relationship(s). Unconscious memories now become accessible.
- **Stage 6**: *Systematic analysis of the transference*. The entire process, including each defence, is repeatedly recapitulated to bring the patient insight into his entire defensive system and the feelings that have been dissociated beneath the resistance.
- **Stage 7**: *Dynamic exploration of the unconscious*. Systematic analysis of the transference furthers access to the unconscious; the patient’s memories of early attachment relationship(s) can now be examined. Meaningful insights and resolution of traumatic memories occur to the degree that the resistance to their examination has been removed (partial, major, complete) through the previous stages.
- **Stage 8**: *Recapitulation, consolidation and treatment planning*. Insights and understanding of how the attachment ruptures led to the development of defences to block the underlying feelings and fantasies, and how and why these defences became activated in the situations in which the patient has been experiencing problems are reviewed.

In summary, ISTDP focuses on the experience of feelings in the here-and-now of the transference. In response, the patient begins to automatically manifest anxiety and defend against dissociated feelings from breaking through into conscious awareness. This enables the therapist to assess the anxiety patterns and defensive processes of the patient in vivo. If necessary, the patient is helped to restructure his anxiety to striated muscles. The therapist continues the central dynamic sequence through one of the two routes to the unconscious, depending on whether the patient is primarily manifesting anxiety or defensiveness in the transference that block the rise of anxiety and the underlying complex transference feelings. Pressure is applied either to experience the feelings that are creating anxiety (if anxiety is in the transference), or to the defences that are blocking the rise of anxiety and complex transference feelings. This directs the resistance into the transference, paving the way for the eventual conscious experience of transference feelings and exploration of the unconscious.

In this paper, we report on the first session between the musician and the ISTDP therapist, which constituted both an assessment and a trial therapy.

#### Case

The participant, Kurt,^1^ was a 55-year-old professional orchestral string player with one of the eight premier state orchestras in Australia. His orchestra was participating in a national study on the mental and physical health of professional classical musicians at the time of recruitment. Volunteers for the trial of ISTDP underwent in-depth interview with the first author and completed the baseline protocol comprising the *Kenny*-*Music Performance Anxiety Inventory* (K-MPAI) and tests of depression and trait anxiety reported elsewhere (see Kenny et al. 2014b). Musicians with scores on K-MPAI that were equal to or greater than 105 were eligible for inclusion in the trial therapy study (Ackermann et al. 2014).

Kurt had been a member of the orchestra for 32 years and had reached the position of assistant principal in his section. Due to the recurrent ill health of the principal, he had been called upon on many occasions to fulfil this role. He distinguished himself in the position, despite experiencing long-term severe music performance anxiety, which he had managed with a range of therapies and self-help strategies, such as daily affirmations, visualizations and meditation. He was expected to win the position when it was finally vacated by the incumbent. However, on the day of the audition, he “fell apart”—which, to most highly skilled, professional musicians means that he did not play at his best. There was no performance catastrophe or breakdown. Nonetheless, he failed to win the position. This experience motivated him to volunteer for the trial therapy study. All sessions were videotaped for later transcription and analysis.

#### Therapy

The second author conducted the therapy and the third author supervised the therapist. ISTDP commences with a “trial” therapy that proceeds through as many of the eight steps outlined earlier as is appropriate for the patient’s level of psychological resilience. During this initial trial therapy interview, the therapist attempts to establish a working relationship with the patient by identifying any barriers to engaging in dynamic therapy, assesses the presence and type of anxiety and defences operating, and establishes the patient’s goals and willingness to explore the underlying emotional issues.

##### Kurt’s position on the spectrum of psychoneurotic disorders following trial therapy

Kurt manifested striated muscle anxiety. He reported that, at times, during performances, he experienced cognitive perceptual disruption; however, he did not manifest any symptoms indicating CPD during the Trial Therapy and therefore was not placed on the Spectrum of Fragile Character Structure. Kurt built a solid “wall” of defences in the transference relationship. His primary defences were passivity, helplessness, rationalization, detachment, and turning in on himself. He responded well to intervention, turning against his defences as he saw their cost. He engaged well with the therapist to gain access to his previously dissociated emotions related to his ruptured attachments. On the Spectrum of Psychoneurotic Disorders, he was moderately to highly resistant.

This first session illustrates the various phases of the therapeutic process.

1. Phase of inquiry

The therapist explored the patient’s presenting problem and ability to respond. It is a dynamic and diagnostic process that provides information about the patient’s location on the spectrum of psychoneurotic disorders and the degree of resistance. The phase of inquiry can rapidly move to the phase of pressure that will continue the enquiry and reveal the nature and level of resistance likely to be encountered.

Th: What are the problems that bring you here? [*Inquiry*]
KURT: The difference between when I practice at home, and when I actually play in an exposed situation seem to be a mountain I can sometimes climb and sometimes not… for some unknown reason it’ll be okay 1 day and not the next… I meditate and I exercise a lot, I look after myself. I don’t drink too much. I don’t smoke… but I can’t control the performance situation.

##### Inquiry moving into the phase of pressure

In this phase, the therapist presses the patient to be more specific, to clarify the meaning of idiosyncratic or vague use of words, so will ask probing questions, and will direct focus onto the actual experience of feelings.

Th: What happens to you in music performance situations? [*Exerting mild pressure by asking for detail*]
KURT: …[T]he mind is the thing that plays tricks with me…
Th: In what sense? [*Pressing for further detail*]
KURT: It will become very active and to a certain extent becomes negative.
Th: Becomes negative? What sort of things is it saying to you? [*Patient continues with vague and avoidant answers*—*therapist continues to press for clarification*]
KURT: It’ll say watch out; it’ll become cautious, over cautious about something and it’ll be very heightened. I guess it’s that fight and flight sensation, feeling that I’m in a very dangerous situation, and if I’m not really careful… things could happen.
Th: Things could happen? [*Maintaining a specific focus to clarify patient’s meaning*]
KURT: Yeah, that… overload my brain… to the point where the things I should be really focusing on don’t get enough attention.
Th: Okay. What happens, physically, in your body? [*Directs attention to experience of feelings in the body*—*diagnostically assessing for striated or smooth muscle tension*]
KURT: That can cause all sorts of funny sensations that… I can’t prepare for when I’m practising. I get funny feelings in my hands.
Th: You get tingling, you mean, or numbness? [*Pressing for specific detail*]
KURT: No, not really. The worst part about it is this feeling of this [touches head] taking over.
Th: So there’s negative thinking? [*Assessing for cognitive distortions*]
KURT: Yeah, yeah. I have high blood pressure, hypertension. I am taking beta blockers, which have helped me to a certain extent to stop trembling.
Th: Okay. So you used to get trembling?
KURT: Yeah. A lot… I controlled it… somehow…but when I’m in the extreme situation where I’ve got to play solos, I get this fear feeling. Lately, I’ve been trying to practice affirmations and visualisations about an upcoming event I’ve got to play for.
Th: When’s that?
KURT: End of October.
Th: Okay. So that’s in 5 weeks? [*Establishing a possible goal*]
KURT: Yeah. Five weeks away, so I’m working on that… It has taken discipline to do it every day; I usually meditate and then I go through this round of affirmations on myself and then try to visualise the scene that I’m going to be walking into to play. I have done some concerts using visualisation and self-visualisation and self-affirmation and it’s worked OK at times. But it’s a 50/50 proposition. I never know if it’s going to do the job.
Th: Okay. But when you’re actually playing in these exposed situations, there’s still negative thinking? [*Indirectly challenging the strategies used to date*]
KURT: There can be.
Th: That’s dangerous. [*Introducing an anxiety*-*laden area using the word “dangerous” introduced by the patient earlier*]
KURT: And the brain can just trip me up.
Th: What happens to you physically?
KURT: Physically, I did an audition a while ago and I lost the normal feeling in my arm. I couldn’t explain it. It was like it was somebody else’s arm—I couldn’t control it.
Th: It was a feeling of complete detachment?
KURT: Yeah.

The therapist continues his inquiry, asking Kurt what other feelings and sensations he experienced in his body during high anxiety performances such as solos and auditions, checking for *somatisation* (“Do you ever get sick in those situations?”) and *cognitive perceptual disturbances* (“Do you ever lose ability like having blurred vision?”). Kurt did not get sick but agreed that he had blurred vision when very anxious. He also gave other indications of *cognitive perceptual disturbance* (“Even if I know a piece or a part really well, it’s almost like my brain sees it on the paper and doesn’t recognize it. The message doesn’t get through”).

Once the therapist has a clearer picture of the presenting problem, he extends the inquiry to other parts of the patient’s life. This history taking will identify the presence and extent of character neurotic process.

Th: Does the anxiety turn up anywhere else? Is it only in performance or do you become anxious in other aspects of your life as well?
KURT: I get anxious in other things.
Th: Where else do you feel anxious?
KURT: [Sigh] I felt anxious at an orchestra meeting the other day…when there’s a lot of people in the room.
Th: So any public situation?
KURT: Yeah, I get anxious.
Th: What do you do to manage the anxiety in those situations?
KURT: I just sit still and shut up.
Th: So you withdraw into yourself? You become passive and withdrawn. [*Identifying and labelling characteristic defences*].
KURT: I withdraw. I’d feel pretty bloody nervous about standing up and talking in front of a group and I’d probably stutter and my brain would click in. …With about five or six people I don’t feel comfortable… I mean I’m not going to jump out a window because of it…
Th: So can you tell me about the last time you were in that situation when you became very anxious in performance? [*Pressure to a specific current example of the presenting problem*]
KURT: That was an audition a month ago. I did all the preparation. I’m always prepared, I always work as hard as I can to make sure the performance pieces are bullet-proof. I’d worked really hard and consistently for 6 months… But, with auditions goes a negative side… I think “What if I flunk?” which I did. I was auditioning for a seat I was already sitting in. I was acting principal—I’d had the job for 6 months and everybody said, “Yeah, yeah look it’s great—I like the way you’re doing it.” But in the back of my mind, when it comes down to auditions you’re standing behind a screen… You come out, you have a piano and there’s a big screen. Behind the screen are about 18 people sitting there evaluating how you’re playing. You have a set repertoire to play and I choked… I didn’t completely fall apart, but I didn’t feel comfortable…
Th: You choked?
KURT: I got to a certain part in the audition that required small motor skills and my bowing forearm didn’t work.
Th: So you lost sensitivity?
KURT: I lost sensitivity, I got through the audition. They told me I did—it’s all anonymous, but the people recognised my playing—said I’d played really well. I thought I’d probably done worse than I really had. A part of my problem was I’d sat behind those audition screens before. I’ve been on the other side, so I knew the whole process. I knew exactly what they were thinking, what they were looking for so I—in a sense going back on it, I had the cards stacked against me before it I walked into the friggin’ room…
Th: In what sense were they stacked against you? [*Continuously clarifying the patient’s meaning*]
KURT: Within myself, I had them stacked against me. They would have done anything to give me the job, because I was doing a good job, people told me and I knew I was doing a good job. But, unfortunately, the job gets open to anybody. I’m a bit older… The guy who got the job is terrific; I sit next to him, he’s fine; I’m glad he got it. But I was slightly disappointed in myself that I didn’t play as well as I could have.

(2) Phase of challenge

During this phase, the therapist increases the pressure, challenging the patient to identify and turn against his characteristic defences. This sets up a conflict in the patient who is simultaneously maintaining his resistance and developing a therapeutic alliance with the therapist to relinquish his resistance. The first breakthrough occurs when the balance between maintaining resistance and unconscious therapeutic alliance with the therapist is tilted toward the unconscious therapeutic alliance. The phase of challenge begins when the patient actively resists the therapist’s attempts to reach the feelings underneath anxiety. The therapist relentlessly points out the patient’s defences, counters the patient’s rationalizations and blocks irrelevant and distracting talk.

Th: You’re glad the other guy got it? [*Clarification with implied pressure*]
KURT: I was disappointed in myself but I’m glad he got it because he deserves it; he’s a good player. I don’t feel any bitterness about that.
Th: But when you were at the audition behind the screen with people judging you, what were the feelings you were having toward them underneath your anxiety? [*Pressure to experience feelings to ascertain if patient is motivated to explore underlying emotional issues*]
KURT: [Sigh (a sign of rising unconscious anxiety)] Well, I’ve thought to myself that some of those guys should be out there doing it too.
Th: So how are you feeling toward them?
KURT: I was slightly disrespectful, I suppose…
Th: What were your feelings towards them? Do you notice you’re becoming anxious as we ask about your feelings? You’re holding your breath right now. [*Monitoring signs of unconscious anxiety and defence*] [KURT nods] It’s interesting isn’t it? When we draw attention to the feelings underneath your anxiety, your anxiety goes up… So what are your feelings towards them underneath that anxiety? [*Continuing pressure to feelings*]
KURT: A slight hostility, I guess [*Notice the ambivalence in the pairing of the word ‘slight’ with ‘hostility’ suggesting the struggle between maintaining the resistance and allying with the therapist to conquer the resistance to experience and express his feelings*].
Th: Yep. How do you experience that hostility toward them? Separate from that anxiety in your body, how do you feel the hostility? [*Pressure to attend to the physical experience of hostility*]
KURT: I feel they’re judging me.
Th: Yeah. But your feeling toward them about judging you is hostility. Now you get anxious as we talk about this. [Kurt: Yeah] So how do you feel the hostility underneath the anxiety, because it’s like that anxiety is trying to sit on top of that hostility right now [*Clarifying the link between anxiety and hostility (anger) and the effect of anxiety*] [Kurt: Mmmm]… and block it and push it down and not let you have this feeling of hostility in your body. But if you push that anxiety out of the way, how do you feel the hostility toward them?
KURT: I don’t know. I don’t quite know how to answer [*Resistance rising to prevent breakthrough of hostile feelings*].
Th: There is a part of you that doesn’t really want you to look at that; that doesn’t want you to actually know that you feel hostility in your body, that would rather you were anxious. [*Clarifying destructive effect of defences*]
KURT: I feel a certain amount of anger because I’ve done the job well for nearly 3 years.
Th: Oh, 3 years, wow.
KURT: Because the guy who had it before was sick all the time so I took over and I did the job well, everybody said so… But when it came down to being judged in an event that is not related to the actual job, I got kicked in the balls… [*Hostile feelings breaking through*—*unconscious therapeutic alliance supporting expression of anger and hostility*]
Th: You were angry with them?
KURT: Angry, yeah, angry with them and angry with the situation.
Th: But angry towards these people judging you? [*maintaining focus on the judges*]
KURT: To a certain extent, yeah.
Th: How do you feel that anger towards them, as you look at it now? Because there is anxiety there now, so how do you feel the anger underneath that anxiety towards these people? [*Pressure to focus on underlying emotion of anger*]
KURT: I’m trying to deal with it, as in that they’re only doing what they had to do.
Th: Okay. So that’s you rationalizing it. [*Clarify the defence of rationalization*]
KURT: I’d be doing the same thing if I was on the audition panel, I guess.
Th: Do you rationalize your feelings a lot? [Client sighs] That was a big sigh there. [*Signs of striated muscle tension*]
KURT: No, I don’t think so.
Th: So the moment you’re feeling angry with them, you get anxious in your body and tense and then you rationalize and make excuses for them? If you don’t do that, how do you feel that anger toward them? [*Therapist does not collude with patient’s denial or rationalization*]
KURT: I try to dispel it… I try to be tolerant because it’s only a—
Th: But if we look at what it’s doing to you? [*Focussing on the negative effect of the defences*]
KURT: Well, I’ve been upset about it, I suppose.
Th: You’ve been anxious and then you go into performances and you have a very similar reaction, I expect? [*Refocusing on the presenting problem*]. [KURT: Yeah]. It’s quite similar in performances to what it was behind that screen. You get anxious, and it affects your ability to play. Underneath that anxiety, we see that you’re angry. [KURT: Yeah] So if we look at those angry feelings rather than you try to dispel them, because these are just feelings in your body, aren’t they? Anger is just a feeling; it’s not an action, yeah? [KURT: Yeah] You see the difference there? [KURT: Yeah] It’s just a feeling. So if we look at that, how do you feel anger toward them, physically, in your body, if you don’t rationalize it?
KURT: I feel tight…
Th: Okay, that’s your anxiety that’s been crippling you, isn’t it? That anxiety cost you that chair. It was not that you weren’t competent, you were 3 years in the job; clearly you’ve got the expertise. It was anxiety that cost you that chair [*Challenge to turn against the defence*]. [KURT: Yeah] So we don’t want to cling to that anxiety [*Supporting the therapeutic alliance in the use of ‘we’*] [KURT: No] So how do you feel the anger underneath that anxiety?
KURT: I guess I feel it’s my fault.
Th: Ah, so that’s another thing you do with anger, you turn it on yourself? [*Clarifying the defence of turning anger in on himself*]
KURT: Yeah, because it was my way of approaching the whole situation.
Th: So, instead of feeling the anger toward them now, you’re turning it back on yourself and saying it’s your fault. [KURT: Yeah] But we know you’re actually angry with them. [KURT: Yeah] So another way you avoid anger is to turn it on yourself and beat yourself up. So far, you’re getting anxious and tense, and you rationalize it. Then you turn back in on yourself and attack yourself? [*Recapitulating the defences he uses to avoid his feelings*] [KURT: Yeah] Now if you don’t do any of those things, if you don’t go to anxiety; if you don’t rationalize it and you don’t turn it back on yourself, how do you feel the anger toward them? [*Challenge the defences and exert pressure to the physical experience of anger*]
KURT: There’s no right answer to this, is there?
Th: Well, in your body there’s a feeling of anger somewhere underneath this anxiety and a part of you is trying to tell yourself that you’re not allowed to be angry [*Pointing out and challenging the resistance*].
KURT: But if I’m angry towards somebody I don’t know how to deal with that. I mean what do I do about it? I can’t go up and biff ‘em.
  [An interchange occurs at this point about the difference between violence and anger and the difference between action and feeling that had been touched on earlier].
Th: At the moment there’s a part of you trying to tell yourself that if you’re angry you’re going to hit someone. But anger’s a feeling. This part of you is trying to make you anxious instead and you either rationalize it or turn it on yourself and beat yourself up [*Therapist is countering faulty thinking i.e. if you feel angry, you will hit someone*]. So physically, how do you feel that anger? [Client squirms with anxiety] [*Refocusing on the pressure to connect with underlying angry feelings*]

This interchange continues for some time, with the patient continually reporting symptoms of anxiety (e.g., “I feel tight”, “my breath gets shorter”, “I feel tense”, “I feel tight in my chest”) rather than the feelings of anger underneath and the location of those feelings in his body. The therapist sums up:

Th: Underneath that anxiety how do you feel that anger toward them? …We’re seeing underneath that anxiety that’s crippled you and damaged you and cost you that chair, is anger, and rather than getting caught up in that anxiety it would be better to look at that anger to try to understand what the anger is about and what’s driving it and see what we can do to resolve it? So how do you feel that anger toward these people for their judgment of you? [*Recapitulation, then pressure to experience physical manifestations of angry feelings*] *H*ow do you feel that anger in your body toward them? [*Maintaining focus and pressure*]
KURT: I feel like I’m not allowed to show it for a start, so it’s really hard for me to get to it.
Th: Okay. So what happens there? …There’s a part of you focused on me right now, in terms of you’re not allowed to show your anger to me—that I’m not allowed to see it. [*Pointing out complex transference feelings*] You want to keep me at a distance and not really let me—[*Pointing out defence of emotional distancing in the transference and its consequences*]. (Client shifts in his chair with anxiety)
KURT: There’s a part of me that wants to do the right thing.
Th: You look and see what you can do to make people happy around you? Do the right thing all the time? So you are trying to perform for me now? [Kurt nods]. But that’s not good for us because we’re here for you. There’s a part of you trying to abandon you and focus on what’s going to be best for me. Does that happen a lot, that you get left to last? You focus on everybody else first? [*Clarifying cost of defence of focussing on the other person instead of himself*]
KURT: It happens a lot of the time, yeah.
Th: Okay, so that’s one of the problems that you have; it’s hard for you to be centre stage in your life?
KURT: I don’t want to be…
Th: You don’t want to be in the centre of your life? You want to be—
KURT: —the centre of everybody else’s life.
Th: Oh, so there’s a part of you that doesn’t want to look after you and respect you? And back you? You are a highly skilled musician looking to get a job that you were going for after you had held it for 3 years. Yet there’s a part of you that’s going, nah, I’ll just go off to the side and let somebody else get the position. (KURT vigorously agrees). So there’s a part of you that really wants to abandon you and neglect you and push you off and make you last? (Kurt agrees). That’s self-destructive isn’t it? (Kurt agrees) That’s trying to come into play here with me, where it’s trying to focus on what I want? But we’re only here for you.
KURT: Do you mean I’m trying to answer the questions to please you? (Th: Yeah) I’ve done it for so long I can’t get out of the bloody habit. (Client shifts in chair anxiously)
Th: You’ve done it for a long time now; that’s a big problem. You’re not really looking after you. Obviously there’s a part of you that wants to look after you because you’re sitting here with me. (KURT: I’m here). Exactly right. So there’s a healthy part that wants to look after you, but there’s also this destructive part that wants to put everybody else first. That wants to rationalize your feelings, or turn them into anxiety, or turn them in on you and beat you up. All of those things are crippling your life, and we can see in that audition that the anxiety that came out of the fear of feeling your anger actually stopped you getting that job, which you’d held for 3 years, so it’s a very destructive system [*Again pointing out and clarifying the system of defences used to defend against hostile, angry feelings and the self*-*destructive consequences of such a system*].
KURT: Mmm. Yes, I see.
Th: How do you feel right now as you look at this system and what it’s doing to you? What are the feelings coming up—[*Test to see if he has turned against his defensive system*].
KURT: Well, when it’s explained like that, I feel hopeless, helpless and hopeless
Th: So there’s a part of you wanting to collapse and abandon you again? [*Pointing out the defence of going helpless*]
KURT: Yeah. Oh, where the fuck do you go from here?
Th: Let’s look at what’s happening inside you. I get a sense there’s some sort of feeling trying to come up? [*Focusing attention on his feelings; encouraging him to take action against helplessness*] (KURT: Mmm). There’s a part of you that wants to avoid me and put a distance between us, to not really let me into these intimate thoughts and feelings that you’re having right now. [*Pointing out his detachment in the transference*] (KURT: Mmm). There’s a part of you that builds a wall and gets detached. [*Highlighting again his emotional detachment in the transference*] (KURT: Mmm). That’s a sabotaging part as well because whilst that wall’s there I’m useless to you because it doesn’t let us into where the real problems are. [*Clarifying the cost of the defence of emotional detachment*] It doesn’t let us resolve them. So there’s a part of you that also makes people useless to you in your life, keeps them at a distance, doesn’t let them be as close as they could be?

This challenge continues for some time with the therapist pointing out repeatedly how these characteristic patterns of interpersonal interaction are evident in the transference, and after seeking further clarification from the patient, in other important relationships in Kurt’s life. The therapist questions, highlights, applies pressure to attend to feelings, and draws attention to the defences and to what the defences are helping the patient to avoid.

Th: These patterns have damaged you. You don’t let people in or allow them to be as close to you as you want them to be. These patterns are in play here with me, trying to abandon and neglect you by putting a wall between you and me. As you look at that, what are the feelings that you’re having at this moment? [*Encouraging client to overcome his defences and experience his feelings*]
KURT: I’m trying to get further inside myself to try and pull out some reasoning or—[*Defences of rationalization and isolation of affect*]
Th: Mmm hmm. If you don’t go to your head, if you stay in your feelings in your body, what are the feelings trying to come up in you right now, as you look at this sabotaging system and what it does to you—neglects you, puts a wall between you and people who are important to you, turns and attacks you, makes you anxious? [*Constant challenge to the defences*] It’s a very destructive system.
KURT: It’s a voice that pops up all the time, and in performances, it’s there.
Th: Yeah, that’s the thing that turns into anxiety, isn’t it? It’s everywhere in your life, but it’s most paramount and prominent in your performances?
KURT: Mmm. So how do I deal with that? [*Defence of passivity: Putting the responsibility for change onto the therapist*.]
Th: That’s a good question, how are we going to deal with this because you’ve been passive in response to it for a very long time. You’ve let this thing roll over the top of you. You haven’t stood up against it. [*Putting the responsibility for change back to the client*] (KURT: No). Why? Obviously you’re highly intelligent, highly skilled, yet you let this thing just roll over the top of you and smash you. [*Continuing to encourage the client to recognise that he has capacity to fight against the destructive defensive system*] (KURT: Mmm). But why? What have you done that you deserve this torture? [*Rhetorical question to the unconscious to try to get it to begin to turn against the punitive superego*]
KURT: I dunno whether it’s environmental or I was born like it. [Client shifts in seat anxiously] [*Rationalizing, avoiding feeling*]
Th: Did you notice there are feelings trying to come up again and that part of you wants to change the subject and dismiss them, and that’s another way to keep a distance from me. [*Challenging distancing manoeuvres in the transference*] (KURT looks down at the floor). You want to avoid me right now so that I cannot see all these feelings that you’re having? (KURT: Mmm). But why do you want to do that to yourself, because you are locking yourself in prison right now. [*Clarifying the cost of the defence of emotional detachment*] Rather than being fully here with me, your eyes want to go to the floor. Rather than sharing your feelings with me you want to put distance between us, and that’s destructive. [*Pointing out the detachment in the transference, with implied pressure to do something about it*].

This interchange continues, adding progressively more pressure to recognize and turn against his defences.

(3) Head on collision

This phase consolidates the challenges to the defensive system that manifest as resistance in the transference. This relentless assault on the defences that maintain the self-sabotaging, self-defeating and self-destructive stance of the patient intensifies the transference feelings and mobilizes the therapeutic alliance. At this point, complex transference feelings erupt—both anger at the pressure to give up defences and appreciation of the therapist’s commitment to work with the patient.

Th: This system wants to detach you and try to make you distant, close you off and find excuses for things, rather than focus on what’s going on inside. But is that really what you want to do? Or do you want to actually understand what’s going on inside you and free yourself from this system? [*Head on collision with resistance. Pressure to his desire to overcome his problems*]
KURT: Of course I do.
Th: So what are your feelings that are coming up inside you right now?
KURT: Not feeling comfortable…
Th: What are the feelings coming up underneath that anxiety? [*Pointing out the anxiety and applying pressure to focus to the feelings under the anxiety*]
KURT: Sadness.
Th: Yeah. There’s sadness there. But part of you says you’re not allowed to share that with me. Why shouldn’t you have your sadness? Why shouldn’t you be here with me and share that with me? Because that’s what we’re here for, isn’t it, to get to the core of these problems? [*Head on Collision with resistance*—*pointing out destructiveness of defences*] (KURT: Mmm).
Th: A part of you wants to cripple you, doesn’t want you to really connect with anyone. [*Continuation of head on collision*] (KURT: Mmm).
Th: What are you going to do about that unless you want that to continue? [*Pressure to do something about the defensive system; returning responsibility to patient*] What are you going to do about that here with me unless you want that distance to continue and that sabotaging system to dominate you? [*Challenge to change behaviour in the transference*]
KURT: Just try and feel real feelings underneath.
Th: Uh-huh, so when your eyes are here with me, what are the feelings that come up in you? [*Eye contact equals intimacy. Applying pressure to feelings that intimacy brings up in him. Intimacy activates the core attachment rupture*]
KURT: I feel pretty exposed and—
Th: And the feelings that come up—
KURT: Wondering what the actual core of me is.
Th: Uh-huh, that’s a good question for us. (KURT: Yeah). There’s a part of you that wants to understand what’s going on deep inside you. What’s the engine that’s driving this anxiety and this destructive system? As you feel yourself here with me, what are the feelings coming up? [*Pressure to feelings in this moment trying to overcome the defences*]. You don’t let that destructive system put distance between us. [*Challenge to defences*]. (Kurt sighs) What are the feelings you’re having under that sigh? [*Pressure*] Your eyes want to go away and you want to settle everything down. [*Pointing out defence of avoidance*] Have you noticed that?
KURT: Mmm, yeah, I know. I pull away sometimes.
Th: It’s a crippling system though, isn’t it? (KURT: Probably) Then there’s that distancing—you have put your hands across your chest. [*Pointing out how he’s building a physical wall between therapist and him*] (KURT: Mmm). This is the part of you that wants to build a wall here that wants to hurt you because it wants to push me out and make me useless to you. [*Head on collision with resistance. Pointing out the cost of continuing the defences*] Then you are left with all this misery and suffering, not performing to your level of skill [*Continuation of head on collision with reference to the presenting problem as a way to motivate the patient to give up his resistances*] (KURT: Mmm). Not living the life you’re capable of, that wants to sabotage and put a wall between you and me and not let me in. But once that wall’s there, I’m useless to you. [*Continuation of head on collision*]

In this phase of head on collision, two separate themes run parallel—(1) not realizing his potential because of intense anxiety and (2) his defensiveness in the transference. These themes are linked: the sabotage, of which the wall is a major component, prevents him from having the life of which he is capable. The head on collision aims to intensify complex transference feelings and resistance to the point where the resistance breaks down, allowing the underlying feelings to be experienced. In the following dialogue, there is a shift away from the defences to an awareness of the sadness underneath the anxiety and anger.

Th: At some point today we come to the end of our time; if we don’t do anything the wall stays there. You’re going to walk out with the same problems you walked in with. [*Continuation of head on collision*]
KURT: I just don’t know what to do—I—
Th: Mmm. There’s that part of you that wants you to go helpless. [*Pointing out defence of helplessness*] It doesn’t want you to let these feelings come out. Yet there’s all this sadness trying to come up in you. (KURT: Yeah). Did you notice that sigh? There’s anxiety and you’re trying to settle everything down and keep all that sadness inside. Why do you want to keep distance between you and me when we’re here to try to get to the core of these problems? [*Continuation of head on collision*].
KURT: I want to get to the core, I don’t know how to get there; I’m lost. [*Despite his helpless stance, Kurt’s unconscious signalling system*—*striated muscle tension and sighing respirations*—*is evidence that Kurt is responding to the head on collision*]
Th: Did you notice how that wall and that distance and that anxiety are designed to keep us away from that core?
KURT: Yeah, I believe that, but I don’t know how to thrust it off—
Th: So how do you feel the sadness in your body at this moment? [*Pressure to physical experience of sadness*]
KURT: I feel tight in the chest and tension in the throat.
Th: Yep, so there’s the anxiety again trying to block everything, trying not to let you have your feelings. (KURT: Yeah, yeah). And the sadness underneath that anxiety, how do you feel that right now?
KURT: I feel angry I’ve got it.
Th: You feel angry? Well, let’s look at that, with whom you are feeling angry?
KURT: I shouldn’t say [*Kurt recognizes the defence from earlier discussion in the session of turning his anger in on himself*] but myself—
Th: So it’s turning in on you again? It’s trying to attack you, to smash you down and not let you feel your sadness. It wants to beat you up for being sad. Now, what are the feelings you are having toward me as I try to understand what goes on inside you?
KURT: [raises his voice] Oh, you’re tearing away at a sore.
Th: So what sort of feelings does that bring up in you toward me as I tear away at this sore?
KURT: You’re not the friendly guy I thought you were. (Kurt shifts in his chair and sighs).
Th: So, what are the feelings that come up in you toward me, as the two of us push toward this core of you? [*The use of the phrase “the two of us” draws Kurt’s attention to the unconscious therapeutic alliance*]
KURT: Not anger, but you’re niggling away at my anger.
Th: Not anger? [*Challenge*]
KURT: OK. You are giving me the shits. [*Acknowledges anger in the transference*]
Th: Makes sense though, doesn’t it? Because I’m pushing, I’m tearing away at your sore?
KURT: Nothing personal [*Distancing manoeuvre*].
Th: But there’s anger toward me?
KURT: You’re getting a bit too close—
Th: Should we look at this, or—
KURT: Oh yeah, you go right ahead—
Th: Okay. How do you feel this anger toward me? It’s good you’re being honest.
KURT: [angrily] I want you to go away; I want you to piss off. I’ve had enough.
Th: Uh-huh. So there’s the anxiety; it wants you to run away, it wants you to avoid? (KURT: Yeah).
Th: But underneath that anxiety?

(4) Moving towards a breakthrough into the unconscious

KURT: When I was a kid, I was bred that kids should be seen and not heard. You’re not allowed to cry or have your own thoughts. You just go outside and shut up. I don’t want to hear about you and all that sort of shit.
Th: Who was that?
KURT: My mother and father.
Th: Both of them?
KURT: Yeah, both of them. So I could hang it on them…
Th: But we’re not looking at blame, we’re looking at understanding.
KURT: Yeah, I’m understanding that—
Th: At the moment there’s anger in you that wants to come up toward me and there’s sadness in you about what this destructive thing is doing to you. That old system of “you’re not to be seen or heard” is trying to crush you rather than let us look at these feelings that have been pushed down inside you since you were a boy. That sadness has just got much stronger. [*Therapist commenting on Kurt’s body language*] (KURT: Mmm).

(5) Breakthrough into the unconscious

After repeated pressure, challenge and head on collision with the patient’s resistance, there is a breakthrough to the unconscious in which the patient himself identifies the source of his emotional pain in his early depriving and abusive relationship with his parents.

Th: Why do you need to fight it now? Why do you need to go to that destructive system instead of giving yourself the freedom to feel?
KURT: Yeah [crying]. [*First breakthrough into the unconscious. There is grief about the defences and grief about his childhood that created the destructive defensive system.*]
Th: There’s a lot of pain and feeling there.
KURT: [crying] I had a really cruel childhood.
Th: Yeah. Let that pain come up here, it’s been trapped there a long time.
KURT: [Crying] I was just abused as a kid, both physically and mentally I was abused and it hurt.
Th: Yeah, let it come up. Don’t crush yourself. Let all that pain come up. You don’t have to crush that or push it back down.
KURT: [crying] I never got listened to; never got understood.
Th: There’s waves of sadness and pain that are coming up inside you… who abused you?
KURT: My mother and father. My father beat me, and my mother was just crazy.
Th: You had a crazy mum.
KURT: She loved me but—
Th: Yeah, she loved you. [*This stirs up more painful feelings*]
KURT: But she was fucking crazy, mad, in and out of the loonie bin.
Th: Mmm hmm, and that was very painful. You don’t need to fight that.
KURT: [Sobbing] oh, fuck…
Th: You can face that now. That’s what you and I are here for—to face this. There’s a lot of pain you’ve been carrying for a long time. (Kurt turns his head and looks at the floor). Did you notice that a part of you is really trying not to be here with me right now? (KURT: Yeah). But we want to get to these feelings, don’t we? Why should you have to carry all this around inside you? [*Pressure to his motivation to deal with his problems*]
KURT: My mother’s dead anyway, but my father’s still alive.
Th: But the feelings aren’t dead. (KURT: No). You’ve been carrying those painful feelings since you were a little boy. Imagine what must happen to you every time you come before an audience. [*Linking presenting problem with painful childhood feelings*]
KURT: Yeah. It’s pretty torturous.
Th: Yeah. The judgments; the abuse. (KURT: Yeah). All that floods through your mind at some level when you stand up to perform. (KURT: Yeah, it does).

Imagine the conflict in a performing artist who has been “bred” to be seen and not heard, and abused for expressing himself!

## Discussion

Kurt was diagnosed with music performance anxiety; subtype 2, although there were elements of subtype 3 in his presentation. Kurt reported generalized social anxiety, particularly in groups, as well as panic that resulted in his sitting still and shutting up, behaviours akin to a “freeze” response seen in extreme anxiety. Although Kurt reported some symptoms of cognitive perceptual disruption (blurred vision) and conversion (his bowing arm felt “like somebody else’s arm”), he did not display these in the therapy setting and was therefore not placed on the fragile character spectrum.

In this 3-h trial therapy of ISTDP, Kurt was able to access anger, guilt, pain and grief regarding his difficult childhood; that is, the emotional content of his attachment ruptures that had been defended against all of his life, but which broke through in conditions of stress, such as musical performances. He was able to make the link between his resistances and destructive defensive system to acknowledge his painful feelings as the source of his anxious musical performances.

The ISTDP therapist constantly tracked the emotional state of his patient, assessing the quality of the therapeutic alliance and the level of anxiety that inhibited exploration of the patient’s habitual defensive patterns. By the time adults come into therapy, their defences are experienced as ego syntonic and when challenged, vigorously defend their defensive systems. The central dynamic sequence was applied repeatedly throughout the trial therapy to gain further access to the core psychopathology. Many of the phases of ISTDP were achieved in this session, indicating that Kurt was a suitable candidate for ISTDP. The patient subsequently successfully completed ISTDP, which resolved his music performance anxiety (Kenny et al. 2014a).

## Conclusion

To date, the treatment of choice for MPA has traditionally been combinations of the behavioural and cognitive therapies (CBT). The caseload from which this case was drawn comprised musicians who had previously attended unsuccessfully for other therapeutic interventions that included CBT, mindfulness-based programs, Alexander Technique, and biofeedback training. Detailed psychotherapy process reports of the application of ISTDP to the treatment of severe music performance anxiety are needed to advance the investigation of suitable therapies for the treatment of severe MPA.

The results of this case study demonstrate that ISTDP is a potentially cost-effective treatment for severe music performance anxiety. The first author is compiling a case series to demonstrate the efficacy of this approach with severely anxious musicians (Kenny 2016; Kenny et al. 2014a; Kenny and Holmes 2015). Further research using designs with higher levels of evidence (case control, cohort and randomized controlled trials) are needed to assess the efficacy of this approach with this population.

## Abbreviations

CBT: cognitive behavioural therapy
CDS: central dynamic sequence
CPD: cognitive perceptual disruption
ISTDP: Intensive Short Term Dynamic Psychotherapy
K-MPAI: Kenny Music Performance Anxiety Inventory
MPA: music performance anxiety

## Footnotes

1 Personal details have been changed to protect anonymity. All participants in this trial had undergone previous forms of therapy without lasting success.
